# Empowering Through Education: Understanding Financial Well-Being Among Low-Income Older Adults

**DOI:** 10.1093/geroni/igaa057.2600

**Published:** 2020-12-16

**Authors:** Sara Powers, Sonya Edwards

**Affiliations:** 1 Benjamin Rose Institute on Aging, Cleveland, Ohio, United States; 2 Empowering and Strengthening Ohio’s People, Cleveland, Ohio, United States

## Abstract

Recognizing the need for and importance of financial capability education among low-income older adults, the non-profit agency, Empowering and Strengthening Ohio’s People (ESOP), implemented an 11-part financial education workshop series with a sample of older adults enrolled in the Senior Community Service Employment Program (SCSEP). Over the course of 10-months, participants attended workshops that covered an array of topics (e.g., budgeting and goal setting, avoiding financial exploitation) and completed a variety of measures aimed at understanding their financial situation and well-being (e.g., CFPB Financial Well-Being Scale, Financial Shocks, Material Hardship, Financial Skill). Results indicated that financial well-being scores significantly increased from the beginning of the workshop series (M=54.49, SD=7.23) to the end (M=58.10, SD=8.10); t(48) = 3.66, p=.001. Discussion will offer insights into the subjective financial experiences of low-income older adults who were actively trying to seek and gain permanent employment to improve their financial situation.

